# *Ebolavirus* Database: Gene and Protein Information Resource for Ebolaviruses

**DOI:** 10.1155/2016/1673284

**Published:** 2016-04-14

**Authors:** Rayapadi G. Swetha, Sudha Ramaiah, Anand Anbarasu, Kanagaraj Sekar

**Affiliations:** ^1^Medical & Biological Computing Laboratory, School of Biosciences and Technology, VIT University, Vellore 632 014, India; ^2^Laboratory for Structural Biology and Bio-Computing, Department of Computational and Data Sciences, Indian Institute of Science, Bangalore 560 012, India

## Abstract

Ebola Virus Disease (EVD) is a life-threatening haemorrhagic fever in humans. Even though there are many reports on EVD, the protein precursor functions and virulent factors of ebolaviruses remain poorly understood. Comparative analyses of* Ebolavirus* genomes will help in the identification of these important features. This prompted us to develop the* Ebolavirus* Database (EDB) and we have provided links to various tools that will aid researchers to locate important regions in both the genomes and proteomes of* Ebolavirus*. The genomic analyses of ebolaviruses will provide important clues for locating the essential and core functional genes. The aim of EDB is to act as an integrated resource for ebolaviruses and we strongly believe that the database will be a useful tool for clinicians, microbiologists, health care workers, and bioscience researchers.

## 1. Introduction

*Ebolavirus* is responsible for outbreaks of severe haemorrhagic fever in humans, and it is endemic in Equatorial Africa. EVD is reported in many countries, and all of them had the origins in Africa, by a way of travel.* Ebolavirus* infections have high case fatality rates of around 42% [1–4]. Ebolaviruses can spread by direct contact with either infected patients or mortal remains of infected individuals [5]. Currently, there is no effective prophylaxis (including vaccine) for* Ebolavirus* infection [6]. Hence, the Centers for Disease Control and Prevention and the National Institutes of Health have classified* Ebolavirus* as a “category A bioterrorism agent” [7, 8]. The* Ebolavirus *group comprises five viruses, namely, Taï Forest virus, Reston virus, Sudan virus, Ebola virus, and Bundibugyo virus [9]. The first Ebola virus outbreak was reported in 2013, which resulted in death of more than 2,622 people, including many health care workers; and as of today, approximately 11,302 people have succumbed to the disease. The number of deaths in the current outbreak is higher when compared to that of all the previous outbreaks combined. Thus,* Ebolavirus* outbreaks represent a major global public health problem [10–12]. The comparative analyses of* Ebolavirus* genomes may result in the identification of highly conserved regulatory regions, which may play important roles in* Ebolavirus* biology. We have developed the* Ebolavirus* Database (EDB), a database exclusively for* Ebolavirus* (the EDB is freely available through the following URL: http://bioserver1.physics.iisc.ernet.in/EDB/). The EDB provides a powerful, user-friendly interface to perform various Boolean searches, sequence and literature based searches. The in-built tools in EDB, namely, BLAST and RNA motif search, can be used to compare the genomes of* Ebolavirus*, and they can also be used to identify the RNA motifs within* Ebolavirus* genomes. The BLAST in EDB consists of set of similarity search programs to perform varied homology searches. It can be used to explore all the Ebola viral genomes. It provides a powerful way to compare the novel sequences with previously characterized Ebola viral genes. The BLAST tool highlights the regions of local alignment to detect relationships among sequences that share only isolated regions of similarity [13]. Functional RNA molecules are involved in numerous biological processes, ranging from gene regulation to protein synthesis. The RNA motif search tool helps in the analyses of functional RNA motifs and elements in ebolaviruses genomes and provides useful information for deciphering RNA regulatory mechanisms in ebolaviruses. The Generic Genome Browser (GBrowse) tool in EDB is a combination of database and interactive web pages. It can be employed for manipulating and displaying the various annotations on Ebola viral genomes [14]. The database is also interfaced with the Jmol plugin to visualize three-dimensional structure of* Ebolavirus* proteins [15]. This feature helps the users to analyze the functionally active proteins associated with* Ebolavirus* pathogenicity. The Ebola virus belongs to* Zaire ebolavirus* species and* Zaire ebolavirus* infections have case fatality rate of 42.2%. In a simple case study using RNA motif search and GBrowse tools in EDB, we find that CpG motif (5′-GTCGTT-3′) is more prominent in* Zaire ebolavirus*, and the GC content is higher in 2,000 to 2,399 positions than any other positions in the genome of* Zaire ebolavirus*. This is one of the most important observations from our case studies. Researchers can make use of EDB to locate interesting and significant regions in both genomes and proteomes of* Ebolavirus*. In addition, EDB provides the detailed information on virulent proteins, reservoirs, epidemiology, pathogenesis, and laboratory diagnosis for* Ebolavirus* infections.

## 2. Materials and Methods

The complete genomes of all known ebolaviruses were obtained from the National Center for Biotechnology Information [16] in Genome Feature Format and FASTA format. The genomes of ebolaviruses in these two formats were added in GBrowse. The proteomes of ebolaviruses were retrieved from UniProt database [17]. We used a relational database management system, MySQL, to store and manage the complete data of EDB.

The EDB was developed using PERL/CGI and PERL/DBI modules and the user-friendly web forms were coded in HTML, JavaScript, and Ajax. Solaris server which is well known for its adaptability, security, and scalability was used to host EDB. The database has been thoroughly checked and validated on different platforms (Windows, Linux, iOS, and Solaris) and works well with different web browsers (IE, Chrome, Opera, and Firefox).

## 3. Results and Discussion

### 3.1. Complex, User-Friendly Search Options

The simple or advanced Boolean-based search tools available in EDB are helpful in exploring the complete annotations of genes/proteins of* Ebolavirus*. In a simple text based search, the users can search for genes and proteins of* Ebolavirus* by entering the gene/protein name in the text box. In gene search, the genes can also be searched by entering their corresponding gene number. The user can also browse for the complete genome annotations of the ebolaviruses. The advanced search option in EDB is used to retrieve the list of proteins localized in a specific cellular compartment. EDB allows the users to obtain the proteins based on clusters of orthologous groups and on specific pattern/profile for the downstream system level analysis. EDB aids the users to obtain the proteins based on their status (reviewed/unreviewed). In addition, the links to* Ebolavirus* related PUBMED literatures are provided in EDB.

### 3.2. Facilitating Sequence Based Motif and BLAST Searches

The most important components in the analyses of gene regulation are the sequence motifs with known biological function [18, 19]. The motifs are typically found nonrandomly in the genome [20]. EDB is interfaced with a search tool, “RNA motif search,” which is used to locate the user specified motifs within the coding sequences of ebolaviruses genomes. The tool accepts a stretch of RNA sequence of different length in IUPAC format and then the tool converts the input sequence into a regular expression. In addition to “RNA motif search” tool, BLAST tool is provided in EDB through which the user can perform the sequence based similarity searches for either protein or nucleotide sequences against a particular or all known ebolaviruses [13, 21]. A major advantage of using EDB is that it makes the user save the results of multiple searches in the hard disk of a local computer either as a text document or as a portable document format file.

#### 3.2.1. Case Study

**Figure 1:**
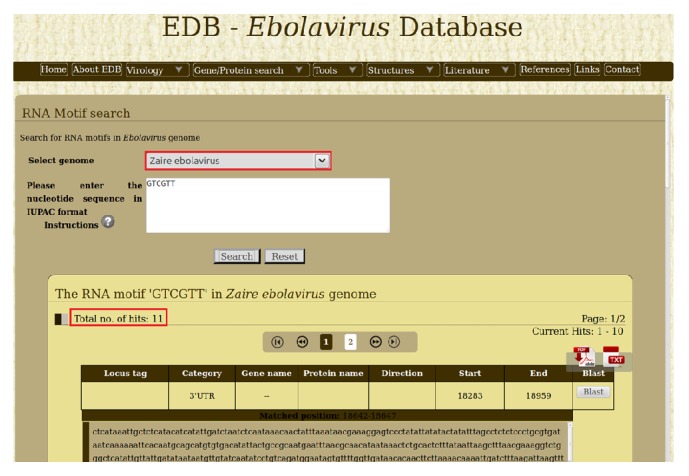
The number of occurrences of CpG motif (GTCGT{2}) in* Zaire ebolavirus* by “RNA motif search tool.”

The CpG motifs are simple dinucleotide sequence of 5′-cytosine-guanosine-3′. The outcome of several studies on immunotherapy of cancer, vaccination, antisense therapy, and gene therapy highlights the importance of CpG motif [22]. The CpG motif 5′-GTCGTT-3′ is identified to be the best stimulatory motif for human cells [23]. The CpG motif was searched against all the ebolaviruses genomes through RNA motif search tool and the results are given in Table 1. Interestingly, we observed that* Zaire ebolavirus* genome has the highest number of occurrences (11) compared to genome of other ebolaviruses (Figure 1). This is just one example of how integration of this tool can lead to new insights while analyzing ebolaviruses genomes.

### 3.3. Genome Sequences Utilizing GBrowse

**Figure 2:**
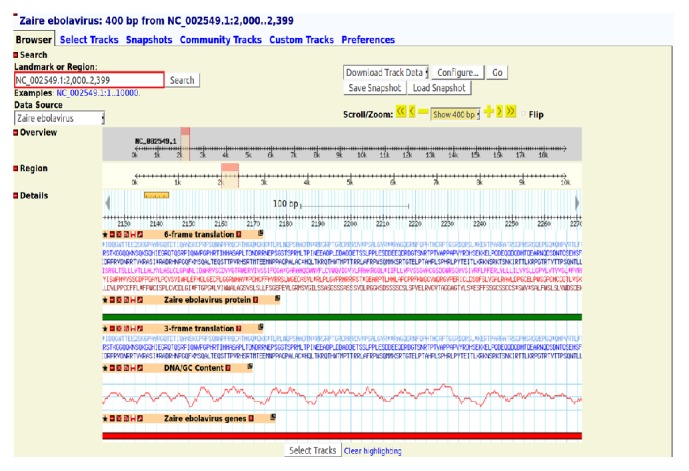
The genes, proteins, GC content, 3-frame translation, and 6-frame translation tracks of* Zaire ebolavirus* from 2,000 to 2,399 in GBrowse.

**Figure 3:**
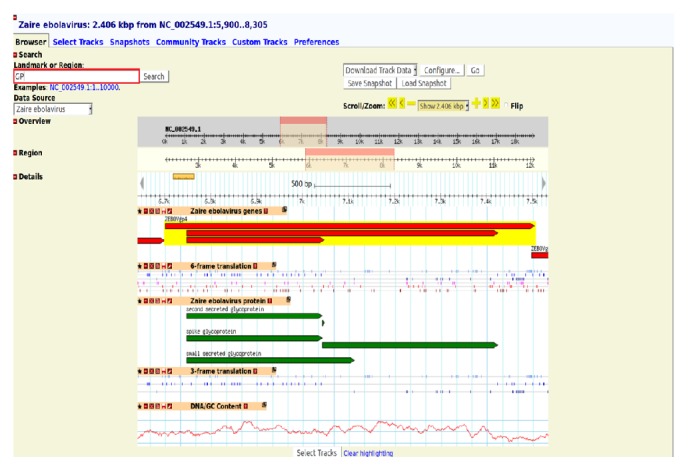
GP proteins of* Zaire ebolavirus* visualized in GBrowse.

The genome content of ebolaviruses has to be easily accessible to researchers for their quick interpretation. To facilitate this, GBrowse developed by Stein et al. [14] has been incorporated in EDB. The browser has special features like navigate, scroll, and zoom in and zoom out over the random regions of the genome. In GBrowse, a specific region of a genome or a landmark can be searched by entering them in the search box available at the top left corner of the page. Then, the browser redirects to the user specified region and displays five tracks: (i) genes, (ii) proteins, (iii) GC content, (iv) 3-frame translation, and (v) 6-frame translation. Each track carries a link to the corresponding information available in EDB or NCBI. Thus, GBrowse enables the user to easily view the genomic content of all ebolaviruses. The genome of* Zaire ebolavirus* (Figure 2) and glycoproteins (GP) (Figure 3) are visualized in GBrowse.

#### 3.3.1. Case Study

The* Zaire ebolavirus* outbreak has resulted in the death of 12,452 persons since its discovery in 1976. The size of* Zaire ebolavirus* genome is 18.96 Kb with 41.1% of GC content [24]. Figure 2 shows various tracks of* Zaire ebolavirus* genome from positions 2,000 to 2,399 where the GC content is found to be notably high.

The only viral protein present on the envelope of* Ebolavirus* is GP. GP is a viral determinant of* Ebolavirus* pathogenicity and probably contributes to haemorrhage during infection [25]. The second secreted GP, spike GP, and small secreted GP are the most important factors responsible for viral entry into the host cell. The coding regions of these GPs are positioned from 5,900 to 8,305 (Figure 3). Interestingly, we found that matrix proteins and minor nucleoprotein are anchored to the sides of these GPs [26]. This viral assembly is unique to* Ebolavirus* and it might help to design effective anti-*Ebolavirus* compounds. Researchers can exploit this option to obtain important features in Ebola viral proteins.

### 3.4. Other Features in EDB

**Figure 4:**
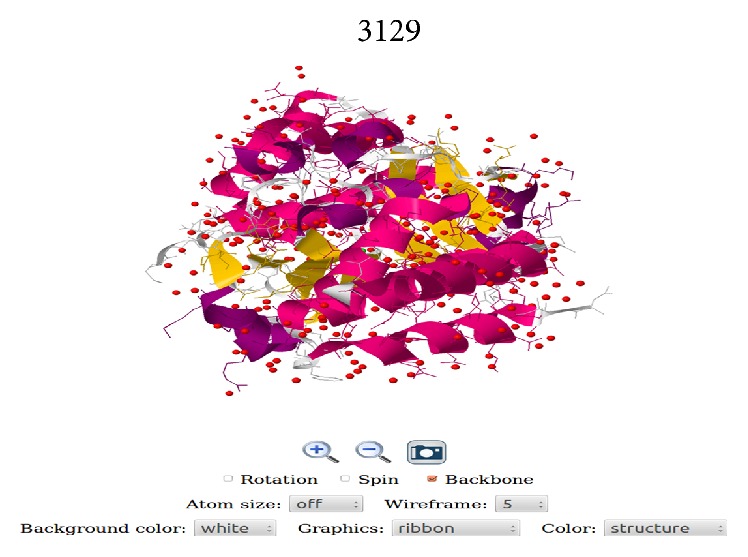
The Jmol view of three-dimensional structure of RNA binding protein (PDB ID: 3L29).

As of March 25, 2016, 63 three-dimensional structures of* Ebolavirus* proteins were available in Protein Data Bank [27]. It is necessary to include these structures in EDB to examine the functionally active proteins. The interactive graphics Java based plugin, Jmol, is incorporated in EDB to visualize these structures.  Figure 4 shows an example of Jmol viewer displaying the three-dimensional structure of a RNA binding protein of* Zaire ebolavirus* (PDB ID: 3L29) [28]. Additionally, the information on virulent proteins, reservoirs, epidemiology, pathogenesis, laboratory diagnosis, and treatment of EVD is provided under “Virology” menu to provide the preliminary knowledge about the ebolaviruses. The links for various resources related to ebolaviruses are provided in the “Links” menu.

## 4. Conclusion

The comparative study of* Ebolavirus* genomes provides insights into the identification of highly conserved regions that play a significant role in* Ebolavirus* pathogenicity. The EDB provides a flexible, user-friendly interface and also offers links to tools such as BLAST and RNA motif search to facilitate the comparative study of* Ebolavirus* genomes. These analyses can be exploited for identifying the putative essential and core* Ebolavirus* genes. We believe that EDB will act as universal single point access of educative and information archive for EVD. EDB will provide valuable genomic and proteomic information on* Ebolavirus* for clinicians, microbiologists, health care workers, and bioscience researchers. The database will be updated on a periodic basis.

## Figures and Tables

**Table 1:** The number of occurrences of CpG motif (GTCGT{2}) in ebolaviruses by “RNA motif search tool.”

Species name	Number of occurrences
*Bundibugyo ebolavirus*	2
*Reston ebolavirus*	2
*Sudan ebolavirus*	7
*Taï Forest ebolavirus*	1
*Zaire ebolavirus*	11
